# A View from the Dark Side

**DOI:** 10.1371/journal.pcbi.0030105

**Published:** 2007-06-29

**Authors:** David B Searls

**Affiliations:** National Center for Biotechnology Information, United States of America

In 1995, when I left a faculty position at the University of Pennsylvania to join the fledgling bioinformatics unit at what was then SmithKline Beecham Pharmaceuticals, the friendly jibes by academic colleagues about “going over to the Dark Side” were, I suspect, only half in jest. There weren't all that many of us practicing bioinformatics at that time, and there was genuine concern that a brain drain to industry might curtail the training of a new generation. Dire talk of “eating our seed corn” made its way into print [1–3].

Other misgivings grew out of the first wave of human genome-wide data then arriving in profusion: the short single-pass cDNA sequences called expressed sequence tags (ESTs) that bubbled up new gene identifications seemingly on a daily basis and that appeared to be suddenly short-circuiting the stately, hierarchical progression of the genome effort through ever-finer stages of mapping while breakthroughs in sequencing technology were labored after [4–6]. The data-management challenges arising from this heady sampling of the genome were making a strong impression, in both the public and private sectors, and the as-yet-unresolved (and highly charged) question of the patentability of genes led to a land rush on intellectual property [7–9]. At the same time a surge of startups with liberal venture-capital grubstakes only increased the demand for skilled gene prospectors [10,11]. All these developments fed concerns that a reckless commercialization of the human genome would be somehow unfairly turbocharged by lavish spending and pure computational horsepower in industry, with legions of apostate academics mining the data.

## Ten Years before the Bench

I had my own concerns in moving to industry, especially as this was my second such excursion. Once before I had departed academia, but for another Dark Side: the computer industry and a job in a central R&D (research and development) function devoted to artificial intelligence (AI). I had joined Burroughs Corporation at the height of the mid-1980s AI frenzy brought on by advances in computer science as well as in hardware speed and capacity, advances that had seemed to leave us on the verge of true “thinking machines.” My reasons then for abandoning the lab and a conventional biology career path had partly to do with the lure of AI at that golden moment when all things seemed possible, and partly with a frustration with what I saw as the slow pace and labor-intensive nature of biological research. Having spent ten years before the bench, and suffering from a bad case of Pipetter's Thumb, I was ready for a computer to do a little of the work. Especially appealing was that rather than having to grow up new bugs from scratch after failed experiments, I could instead just debug and recompile. To me, computers were wonderful effort amplifiers.

Arriving at Burroughs in 1985 did engender some culture shock, but I found the pace at which decisions were made and put into effect bracing after the glacier-paced turnaround times of the public funding apparatus. Of course, the savings in time and futility of grantsmanship were roughly offset by expenditure of time and futility in meetings and corporate bureaucracy. Also, while the decisions could come with breathtaking speed, they could still go either way, and what was more sinister, could arrive unbidden and unanticipated. At times one longed for the lengthy time constants and highly structured expectations of academia, though on the whole I still favored velocity over inertia.

That was during the high summer of AI, which saw not only a boom in research but also in commercialization, with both hardware and software startups galore. The president of the American Association for Artificial Intelligence (AAAI) exulted that “it is clear . . . that our field, perhaps together with molecular genetics, will be society's predominant scientific endeavor for the rest of this century and well into the next. . . .” [12]. With the general buzz around AI, and good support for pushing the envelope from a computer company with deep pockets, what I did in industry seemed qualitatively akin to what I had done in academia. Applied research was intertwined with basic research, with the latest advances being brought to bear on “real-world” problems in what seemed a most felicitous combination [13,14]. It was the AI startups, rather, that seemed relentlessly product-focused and technologically conservative, for the sake of making their next payroll. (It should be noted, though, that one of the more adventurous such companies at the time was IntelliCorp, which had recently changed its name from IntelliGenetics—one of the first serious bioinformatics companies and briefly the home of GenBank [15].)

## A Disturbance in the Force

Perhaps such synergy of scientific advance with commercial application was too good to last. Several inauspicious trends converged as the 1980s drew to a close. First, the mainframe computer business model was rapidly obsolescing, spurring Burroughs to merge with Sperry to form Unisys—thereby breeding what many viewed as just a larger dinosaur. The parent companies had a proud history of advancing computer design, but computing evolved to where what is remembered are just a few vestigial factoids: that Larry Wall hacked together the Perl scripting language—fundamental to the development of the Web and bioinformatics—while holding down a day job at Unisys [16], and that the LZW data compression algorithm was invented by a research group there [17]. The patent for LZW, a basic computing methodology used in several popular graphics formats, made Unisys a bête noire to Open Source free spirits for years and inflamed the debate about whether it should be possible to patent something as “natural” as an algorithm [18].

This was my first experience with the unnerving corporate phenomenon of the mega-merger. In the field of AI, Burroughs had stressed R&D while Sperry had focused on commercialization, which meant that I rather abruptly went from thinking about knowledge representation and logic programming to following sales of LISP machines and turnkey expert system shells (in both cases, products acquired from startups, including IntelliCorp, and relabelled). To further complicate matters, at the same time that the computer industry was having to realign itself, and its appetite for basic research was diminishing for similar reasons, the so-called AI Winter set in. Seemingly overnight the field was perceived as an overhyped disappointment and thus receded from the clamorous marketplace and the glare of the public eye back into the quieter, shaded groves of academe. Attendance at the annual AAAI conference, which had shot up from fewer than 1,000 at the beginning of the decade to more than 5,000 in the mid-1980s, slumped to about 2,000 by the early 1990s, even as the sprawling vendor displays gradually disappeared; it has since settled back to a respectable steady state of about 1,000 core academics reporting steady if relatively unheralded progress [19,20].

Just as AAAI attendance figures and corporate R&D budgets declined, the genome project was ramping up and creating an unprecedented demand for computational support. A cynic might observe that this was a convenient time for me to rediscover my roots in molecular biology, though in fact I had already been working for several years to set up a Biomedical Knowledge Systems group at Unisys, and with my late colleague Chris Overton managed to attract several US National Institutes of Health grants as in-house resources dried up. Now, as the strictures of an industry struggling to reinvent itself closed in, a gap seemed to open between the science and the business, between the pure and the applied, that had never troubled me before. I couldn't disagree with business decisions geared to survival and made many of them myself, but what had before seemed an easy alliance of creative push with strategic pull was now coming apart. Meanwhile, the immense scientific promise of large-scale genome sequencing and its attendant computational challenges beckoned.

## Once More unto the Bench

So it was that in 1991, an offer from the University of Pennsylvania (Penn) to join their faculty and to help build a genome center and informatics lab proved irresistible. Chris and I moved as a team and eagerly set up shop. Almost immediately, the peculiar institution of academia threw up challenges I had nearly forgotten; for instance, there was the matter of academic appointments, which proved problematic for hide-bound committees that couldn't readily countenance a hybrid enterprise like bioinformatics [21]. Did we really belong in a biology department or in computer science? Could much-needed support to bench scientists be reconciled with original computational research? By which standards and conventions should our career development be judged? (Accommodating the hybrid nature of informatics was especially important to me with my research interest at the time in macromolecular linguistics, which spanned the range from formal language theory [22] to practical DNA parsing applications [23].) And, of course, it was back to the treadmill of churning out grant applications. Paylines were then at one of their periodic low ebbs—though liberal in comparison with the current situation—and while I managed to keep several balls in the air successfully, raising money soon occupied vast swathes of my time.

Still, it was a remarkable era for bioinformatics. Crucially, the new discipline was proving its versatility, as the demands of the genome project required a diversification from its early algorithmic focus on efficient string comparison and pattern matching to the combinatorial concerns of genetic and physical mapping, sequence assembly, and so forth. (These are phases of the development of bioinformatics to which I like to refer as Linear A and Linear B, respectively.) In major projects, bioinformatics gradually moved from an ancillary role to a central one, and institutions followed, albeit with a time lag. At the same time, the infrastructure of a true scientific discipline began to coalesce: new and more respectable journals, textbooks, programs of study, and so forth. Larry Hunter, Jude Shavlik, and I drew on the AI tradition of bioinformatics in organizing the first ISMB (Intelligent Systems for Molecular Biology) meeting in 1993, which attracted a little over a hundred people [24]. It grew year by year to become the premier conference in the field (with RECOMB [Research in Computational Molecular Biology], which pulled in the mathematical and algorithmic family branches), now drawing up to 2,000 attendees and attendant flashy vendor displays. The early ISMB and RECOMB meetings also gave rise to the ISCB (International Society for Computational Biology), so that with vigorous conference series and a scientific society, the field had suddenly grown up [25]. Truly, it seemed like a Bioinformatics Spring.

## “That's No Moon . . .”

It was on this upswing that a new opportunity presented itself to me, at SmithKline Beecham (SB). The company had spent $125,000,000 (a lot of money at the time) on a deal to obtain a data stream of ESTs and now wanted to augment the small group managing that data to develop a science-driven bioinformatics function to leverage the investment [11]. I was offered “unlimited” headcount to build a unit (with the obligatory tag “world class”) that would move SB into the forefront of bioinformatics.

The decision to move to industry in 1995 was more difficult than it had been a decade earlier. Academia was rapidly warming to the field, and was finding ways to accommodate it with formal interdisciplinary programs, cross-departmental institutes, and even dedicated departments. But again, this loomed as a long, incremental process—what clinched it for me was the opportunity to do things on scale, to design and build an enterprise, dedicated in purpose but able to turn on a dime, all at once rather than a grant at a time.

SB was as good as its word, and indeed the primary impediment to growth was the difficulty of finding qualified staff in those early days [1]. Year by year my group doubled and doubled again. While there was criticism of SB and other companies for sponging up so many informatics workers, in fact this was a minor effect compared with the rate of maturation of the field as a whole. The draw from industry did far more good than harm in validating the importance of the discipline and establishing the economic demand, to which academia rapidly responded with a supply of both science and trainees. Chris Overton used to say with a wry smile that my leaving was the best thing that ever happened to bioinformatics at Penn (a statement I chose to interpret in a positive light).

## *Plus ça Change* . . .

Today, industry demand has long since reached a steady state; what is remarkable is that it has not appreciably declined. No longer does informatics have the star power to draw feverish investment by pharma, but rather it has become part of the backdrop of discovery—an essential part, to be sure, but not a supernova in itself. There have been many cases of pharma technologies, such as combinatorial chemistry or rational design, that flared briefly (perhaps fed by a bit too much hype) and then subsided back into a contributing role—never quite revolutionizing drug discovery from the ground up, but certainly becoming part of the constellation of tools. Informatics was fortunate, I think, to hit a peak just as it hit its stride in industry, one that was sustainable by virtue of its becoming foundational. By contrast, the burst of bioinformatics startups in the 1990s largely came and went, generally failing to gain traction, at least as a “pure play.”

A key reason for this staying power in industry has been that attribute of versatility. After the “linear” phases of bioinformatics, supporting ESTs and genome sequencing, the “omic” phase drove development of methods to deal with high-dimensional data. Not being tied to any particular technology platform, informatics groups were able to adapt with aplomb. Now the systems phase is driving new modeling methods and network views, and again informatics is making itself not only useful but crucial, particularly with its integrative powers as data sources explode in both volume and diversity. Industry informatics groups have continuously reinvented themselves and largely thrived—provided that they are not pigeonholed into strict functional roles but are allowed to adapt, just as their academic cognates are well-suited to do over time. Ironically, it may have been the standalone informatics vendors who were most disadvantaged by the shifting data and technology landscape, in that they had to commit to extended product lifecycles that simply could not keep pace, even with the public domain.

## Déjà Vu All over Again

Having felt the chill of the AI Winter and the decline of mainframes, I developed an exquisitely sensitive built-in déjà vu meter, which pinned in 2000 when SB and GlaxoWellcome merged to form GlaxoSmithKline (GSK). Each of the parent companies had themselves been formed by mega-mergers within the previous decade, and their combination now formed the second-largest pharma worldwide. By chance, each had invested relatively heavily in internal informatics capabilities, and so this time the groups were more alike than different and their cultures combined fairly smoothly and to good effect. Not all mergers are created equal, and this one has worked well, but there is no denying the fact that they are a lot of work and that these recurrent paroxysms of industry are in marked contrast to the organizational stability of academia. Mergers and major reorganizations are just the most obvious manifestations of the relatively more rapid adaptations that characterize the commercial side, for better or worse.

However, the constant struggle to optimize organization in industry is periodically mirrored in academia, precisely at the point at which scientific disciplines evolve. When I first arrived at Penn there was no comfortable home for the new interdisciplinary field of bioinformatics, but academia adjusted, finding new ways to bridge that particular divide between molecular biology and engineering [26]. That same divide abides in industry, where IT (information technology) groups are traditionally functionally distinct from the scientific lines. Where to put informatics? In my 12 years in this particular business, I believe I've managed groups in nearly every possible configuration, starting my pharma career in the IT line, then moving to the discovery organization, at one point running a bioinformatics function separate from cheminformatics, then combined; and with either a full-service scientific and engineering remit or, most recently, with one focused exclusively on the scientific/analytical function. Each architecture had its strengths and weaknesses, depending on the wider context, and to this day I couldn't swear one is superior overall. Whatever the org chart says, what is essential is that the informatics be embedded with the bench science and that the engineering be embedded with the informatics, in an intimate association with minimal “starting friction” and an appreciation on all sides of the difference between traditional IT and scientific computing.

Thus, far more important than the organization of departments into lines (or schools) is the effective communication and interaction among them—what is called working in the matrix—and this is where pharma excels, even over other industries. Drug discovery by its nature is a series of temporal and functional handoffs, but these can only be effective when there is both continuity and, at any given point, the collective application of multiple interdependent skill sets. Academia tends to accomplish this through ad hoc collaborations and exchanges of reagents (what we used to call “clone by phone”), or on a larger scale by establishing interdisciplinary centers and institutes with cross-appointments of faculty; pharma does it as a matter of course. On any given project team, the range of scientific specialties arrayed against a problem is fascinating to behold, the more so because it is taken for granted, as an unremarkable aspect of doing business. Thus informatics, a quintessentially interdisciplinary pursuit, assumes a very natural place in the matrix of pharma. Moreover, its versatility allows it to keep pace with the rate of technological change embraced in industry, while organizationally mirroring the continuous reinvention of the field as a whole.

## The Matrix Revolutions

Pharma is facing many external pressures in the present age, economic and otherwise, all readily apparent in the news media; one response to this is an increasing virtualization of the industry, by which formerly monolithic and largely self-contained R&D enterprises are increasingly outward-facing. This trend to virtualization takes many forms, from offshoring of support activities, to outsourcing of key processes, to inlicensing of assets, to academic collaborations, to precompetitive consortia—all with an increasingly global reach. In truth, pharma has always depended to a significant degree on academia for the most exploratory new science and on startups for the riskiest new technology, so the change is more quantitative than qualitative. But for an itinerant like me, it's gratifying to see that the economic drivers of this industry not only call for a wonderfully stimulating smorgasbord of skills, but for the continuous exchange of science, technology, and people too. It's hard to imagine that dynamic disappearing under any future model of pharma, whatever the economic pressures and however radical the changes. Informatics is not a dalliance for pharma, it is ingrained now in the process. At the same time, pharma by its integrative nature provides the major economic exigency for informatics technology.

In short, I no longer worry about an Informatics Winter, even with chill winds about—there's light on the Dark Side. 

## Abbreviations

AAAI: American Association for Artificial Intelligence
AI: artificial intelligence
EST: expressed sequence tag
GSK: GlaxoSmithKline Pharmaceuticals
ISMB: intelligent systems for molecular biology
IT: information technology
Penn: University of Pennsylvania
R&D: research and development
RECOMB: research in computational molecular biology
SB: SmithKline Beecham Pharmaceuticals
